# Genome-Wide Identification and Expression Analysis of Sugar Transporter (ST) Gene Family in Longan (*Dimocarpus longan* L.)

**DOI:** 10.3390/plants9030342

**Published:** 2020-03-08

**Authors:** Ting Fang, Yuan Peng, Ya Rao, Shenghao Li, Lihui Zeng

**Affiliations:** 1 College of Horticulture, Fujian Agriculture and Forestry University, Fuzhou 350002, China; fangting@fafu.edu.cn (T.F.); pengyuan456@outlook.com (Y.P.); 18188317868@163.com (Y.R.); MAYJULY807@163.com (S.L.); 2 Institute of Genetics and Breeding in Horticultural Plants, Fujian Agriculture and Forestry University, Fuzhou 350002, China

**Keywords:** sugar transporter, *Dimocarpus longan*, flower bud differentiation, alternative splicing, soluble sugar

## Abstract

Carbohydrates are nutrients and important signal molecules in higher plants. Sugar transporters (ST) play important role not only in long-distance transport of sugar, but also in sugar accumulations in sink cells. Longan (*Dimocarpus longan* L.) is one of the most important commercial tropical/subtropical evergreen fruit species in Southeast Asia. In this study, a total of 52 longan sugar transporter (DlST) genes were identified and they were divided into eight clades according to phylogenetic analysis. Out of these 52 DlST genes, many plant hormones (e.g., MeJA and gibberellin), abiotic (e.g., cold and drought), and biotic stress responsive element exist in their promoter region. Gene structure analysis exhibited that each of the clades have closely associated gene architectural features based on similar number or length of exons. The numbers of DlSTs, which exhibited alternative splicing (AS) events, in flower bud is more than that in other tissues. Expression profile analysis revealed that ten DlST members may regulate longan flowerbud differentiation. In silico expression profiles in nine longan organs indicated that some DlST genes were tissue specificity and further qRT-PCR analysis suggested that the transcript level of seven DlSTs (*DlINT3*, *DlpGlcT1*, *DlpGlcT2*, *DlPLT4*, *DlSTP1*, *DlVGT1* and *DlVGT2*) was consistent with sugar accumulation in fruit, indicating that they might be involved in sugar accumulations during longan fruit development. Our findings will contribute to a better understanding of sugar transporters in woody plant.

## 1. Introduction

Carbohydrates are the main components that not only provide energy sources and building blocks for cell but also constitute osmotic and act as signaling molecules throughout the physiological process and corresponding both abiotic and biotic stress responses in higher plants [1]. Additionally, two kinds of carbohydrates related to sugars, polyols and organic acids, have complementary roles in plant growth and development. Furthermore, the composition and content of sugars is a key factor to determine fruit quality, and impacts on the overall organoleptic quality and flavor of fruit, especially sweetness because different kinds of soluble sugars show different relative degrees of sweetness [2].

In plants, sugars are the main products of photosynthesis, which are synthesized de novo in leaves (source) and translocated into fruits, roots, and stems (sink) to supply the carbon substrate for plant growth and/or storage. Consequently, sugar transportation is important to maintain the balance between source and sink [3]. Sucrose is the main transport sugar in the phloem in many plants [4,5]. However, polyols can also be transported sugar in some species [6].

In plants, sugars are mainly stored in vacuoles, which occupies up to 90% of the plant cell volume [7,8]. The composition and volume of the vacuole are determined by the coordinated activities of tonoplast-localized transporters and channels [9]. Movement of sugars from source to sink cells requires multiple transporters.

To date, various sugar transporters have been identified in fungi, animals, humans and plants [10,11,12]. In plants, sugar transporters are divided into three major types: sucrose transporters (SUTs), also called sucrose carriers (SUCs), monosaccharide transporters (MSTs), and sugars will eventually be exported transporters (SWEETs) [13,14]. In addition to transporting sugar, sugar transporters also play an important role in plant growth and development [15,16,17]. SWEETs represent a new class of sugar transporters belonging to the MtN3-like clan and are characterized by seven transmembrane domains (TMDs) [13]. So, the SWEET gene family will not be included in this study.

SUTs and MSTs, which contain the sugar_tr domain (PF00083), belong to the major facilitator superfamily (MFS), which usually contain 12 transmembrane (TM) domains. The SUTs belong to a rather small protein family and are originally group into five subfamilies, including one dicot-specific (SUT1), two monocot-specific (SUT3 and SUT5), and two monocot and dicot (SUT2 and SUT4) [18]. However, the MSTs are more diverse and they are separated into seven subfamilies: (i) the early response to dehydration 6-like (ERD6-like), which is also called the sugar facilitator protein family (SFPs); (ii) the plastidic glucose translocator family (pGlcTs); (iii) the inositol transporters family(INTs); (iv) the tonoplastic monosaccharide transporters family (TMTs); (v) the polyol/monosaccharide transporter family (PMTs); (vi) the vacuolar glucose transporters family (VGTs); and (vii) the sugar transport protein family (STPs). The ST gene family has been identified in several plants, such as *Arabidopsis* [19,20], rice [21,22], tomato [20], grapevine [23], woodland strawberry [24], pear [25], apple [26] and Chinese jujube [27].

Longan (*Dimocarpus longan* L.), which belongs to the family of Sapindaceae, is an important commercial tropical/subtropical evergreen fruit species with a large number of the production in Southeast Asia and Australia [28]. In particular, the acreage and production of longan in China ranks as first in the world [29]. Sugar content is regarded as a key factor to determine the longan fruit quality. Therefore, one of the major goals of longan breeding is to obtain high sweetness cultivars. Up to now, most studies just focused on sugar composition and content in different longan varieties [30,31], while sugar transporter genes have not been studied in longan.

The present study reports on the identification of putative genes encoding STs in longan genome, together with phylogenetic, structural, conserved motifs, cis-elements analysis. Moreover, RNA-seq databases were used to identify alternative splicing (AS) events and expression profiles in different longan varieties and tissues. We also investigated the expression pattern of 23 selected *DlSTs* during fruit development using quantitative real time polymerase chain reaction (qRT-PCR). These results may contribute to understanding the diverse functions of ST genes in longan growth and development.

## 2. Results

### 2.1. The Dynamic Change in Soluble Sugars Concentration During Longan Fruit Different Development

**Figure 1:**
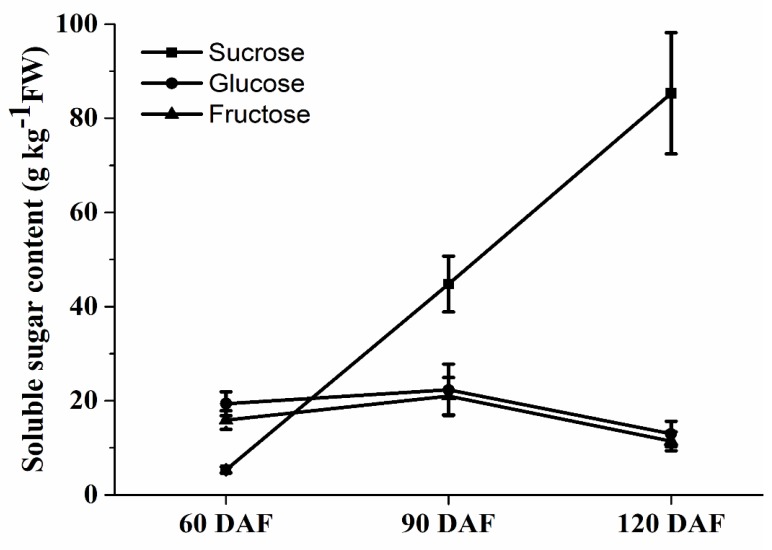
The content of sucrose, glucose, and fructose during longan fruit developmental stages. Values presented as mean ± standard error (SE) (*n* = 3).

The content of sucrose, glucose and fructose had different trends during longan fruit development (Figure 1). The content of sucrose was increased rapidly during fruit development, with concentrations ranging from 5.3 to 85.3 g·kg^−1^ fresh weight (FW). The sucrose content increased approximately 16-fold from 60 DAF to 120 DAF. However, the concentrations of glucose and fructose has a slightly increase from 60 DAF to 90 DAF, then slightly decreased from 90 DAF to 120 DAF. The content of sucrose was higher than glucose and fructose after 60 DAF. On the whole, sucrose is the main soluble sugar accumulated in mature longan fruit.

### 2.2. Identification and Phylogenetic Analysis of the Sugar Transporters in Longan

A total of 52 sugar transporter (ST) genes were identified and they were renamed according to previous research in *Arabidopsis* (Table 1). Amino acid residues of 52 full-length longan ST proteins ranged from 378 (DlSFP8) to 741 (DlTMT1) and the transmembrane domains number varied from eight (DlSFP8) to 14 (DlSUT6 and DlSFP4).

**Figure 2:**
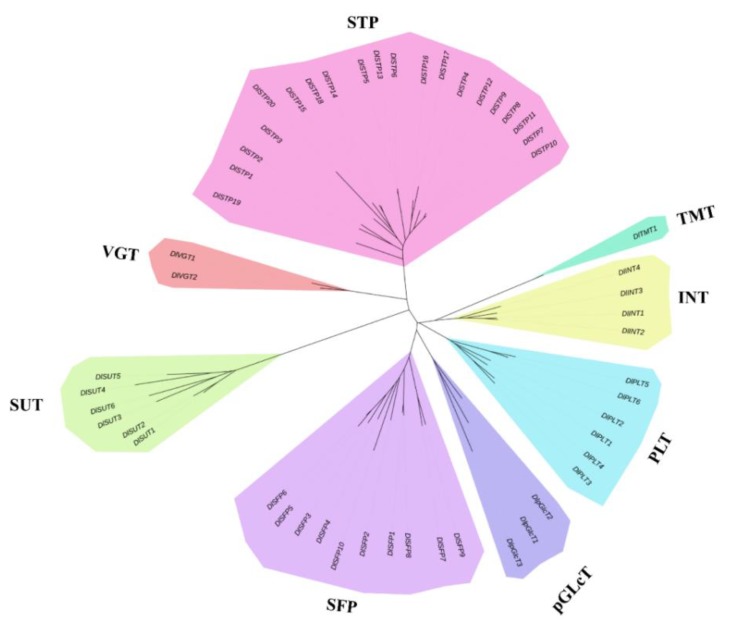
Phylogenetic analysis of DlST proteins. The eight classes are marked by different colors.

To study the evolutionary relationship of the DlST proteins, a neighbor-joining (NJ) tree was constructed using the amino acid sequences of 52 DlSTs (Figure 2). The phylogenetic distribution indicated that the sugar transporter gene family in longan can be grouped into eight different clades. The TMT clade contains only one member, whereas the STP clade contains 20 members. Moreover, ten, six, six, four, three, and two *DlST* genes were annotated as SFP, SUC, PLT, INT, pGlcT, and VGT clades, respectively (Table 1). Furthermore, we compared the clade member numbers among longan, *Arabidopsis*, pear (*Pyrus communis* L.), woodland strawberry (*Fragaria vesca* L.), rice (*Oryza sativa* L.), grape (*Vitis vinifera*), chinese jujube (*Ziziphus jujuba* Mill.), tomato (*Solanum lycopersicum L*.) and apple (*Malus domestica*) (Table 2). Interestingly, STP and SFP display the largest clades in longan, which was consistent with previous reports in *Arabidopsis*, strawberry, grape, Chinese jujube and tomato.

### 2.3. Gene Structural and Conserved Motif Analysis of the Longan DlSTs

**Figure 3:**
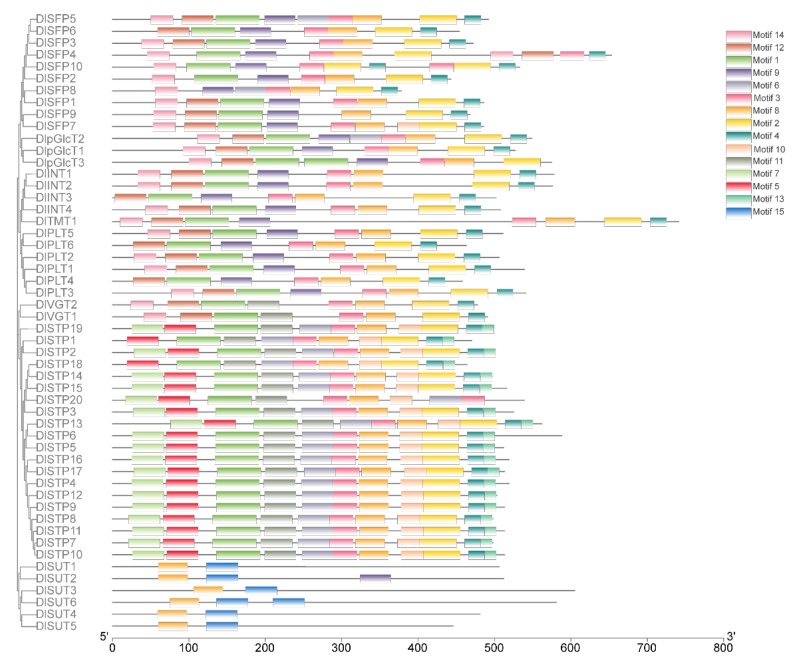
Compositions of the conserved protein motifs of the DlST genes from longan. The sequence information for each motif is provided in Table S1.

To further investigate the characteristic regions of DlST proteins, MEME software was used to analyze the motifs of 52 DlST proteins (Figure 3 and Table S1). A total of 15 motifs were identified in DlST proteins, and motif 8 exists in all 52 DlST proteins, indicating its significance for longan sugar transporter proteins. Five distinct motifs, motif 5, 6, 7, 10 and 13 exist only in 20 DlSTP proteins, suggesting that they may be necessary for STPs. Furthermore, although SUT clade and MST clade had the same functional domains (ST domain), the conserved motifs between them are quite different (Figure 3), indicating a functional difference between SUTs (transport sucrose) and MSTs (transport monosaccharide). Even though MST members had five distinct motifs, the motifs located on STP subfamily are different from other MST gene family, for example, motif 5, 6, 7, 10, 11 and 13 are special for this subfamily.

**Figure 4:**
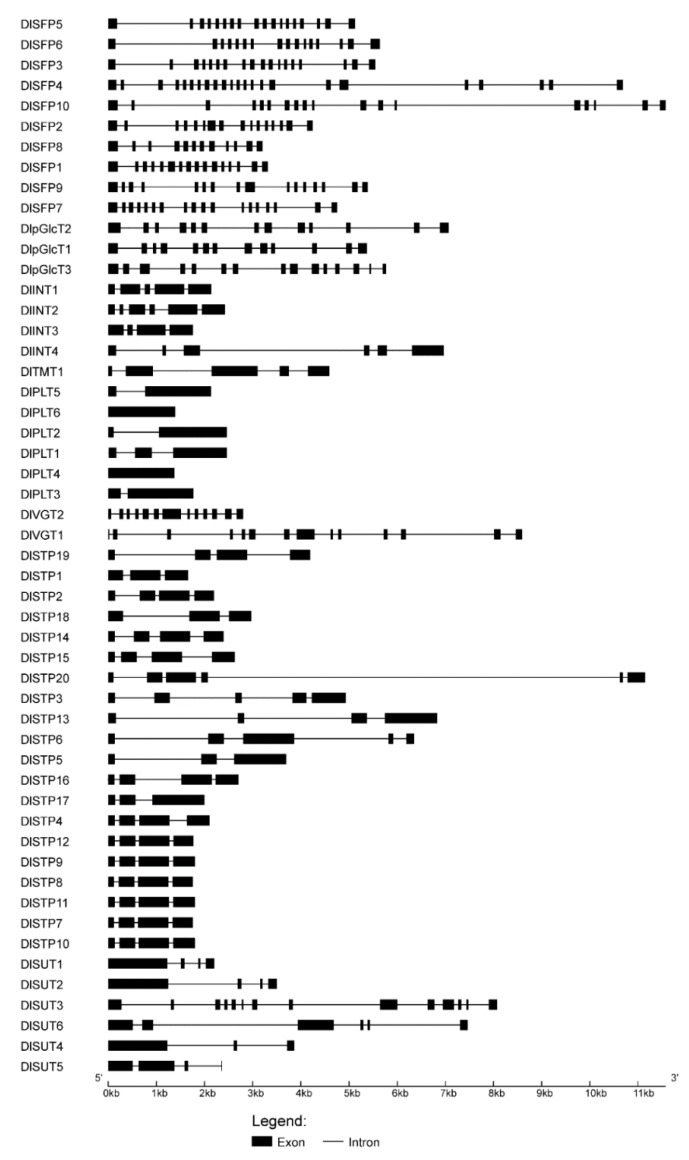
Gene structures of 52 DlSTs.

Gene structures analysis showed that all DlST genes contained one or more exon, varied from one to 23 and different subfamilies contained different exon numbers (Figure 4). For instance, *DlSFP*, *DlVGT* and *DlpGlcT* gene families have more than 11 exons, however, *DlINT*, *DlPLT*, *DlTMT* and *DlSTP* gene families have less than seven exons, suggesting that the number of exons may increase or decrease during evolution of DlSTs, leading to a functional diversity of closely related DlST genes. Overall, all DlST genes showed a closely gene structure because gene members have similar exon numbers or exons length within the same subfamily.

### 2.4. Cis-Acting Elements in Longan ST Promoters

**Figure 5:**
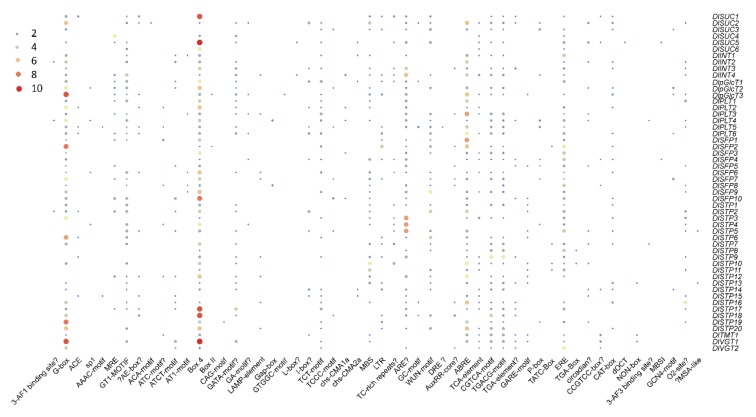
Heat map of the number of *cis*-elements in DlSTs promoter. Color bars and circle sizes indicate the number of cis- elements.

The *cis*-elements, which distributed in gene promoter, may reflect the potential function of genes. Thus, we analyzed all DlST promoters (2000 bp upstream coding sequence) using PLACE (https://www.dna.affrc.go.jp/PLACE/?action=newplace) (Figure 5). As a result, 55 types of *cis*-elements were discovered, including 27 light responsive, 10 plant growth, seven stress responsive, and 11 plant hormone responsive elements. In addition, the light responsive elements took possession of the largest members of all elements, particularly the BOX 4 element, which existed in all 52 DlSTs. In the group of plant growth, the O2-site (zein metabolic regulation) and the CAT-box (meristem expression) were mainly identified. Furthermore, many plant hormone and stress responsive elements were also identified. For instance, TGA-element and GARE-motif are involved in auxin-responsive, TGACG-motif and CGTCA-motif are involved in MeJA responsiveness, GARE-motif and TATC-Box are involved in gibberellin-responsive, DRE is involved in dehydration, low-temp, salt stresses, MBS is involved in drought inducibility. Overall, 130 ABRE and 54 WUN-motif were found in the promoters of DlSTs, indicating DlSTs may play role in respond to ABA and wound-responsiveness in longan plants.

### 2.5. Alternative Splicing (AS) Events Analysis of DlSTs

**Figure 6:**
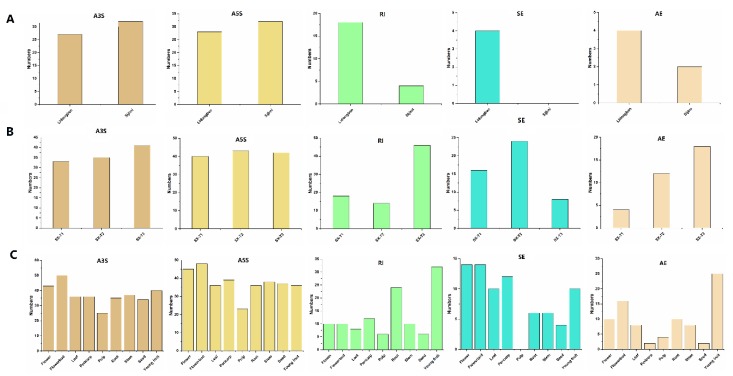
The numbers of different types of alternative splicing (AS) events of the DlST genes. (**A**) The AS events of the DlST genes identified in ‘Lidongben’ and ‘Sijimi’. (**B**) The AS events of the DlST genes identified in different floral bud development stages in ‘Shixia’. SX-T1 represent stage before the emergence of floral primordia, SX-T2 represent stage of the appearance of red dot, and SX-T3 represent stage of the appearance of the first inflorescence apical buds. (**C**) The AS events of the DlST genes identified in nine tissues of ‘Sijimi’ clutivar.

**Figure 7:**
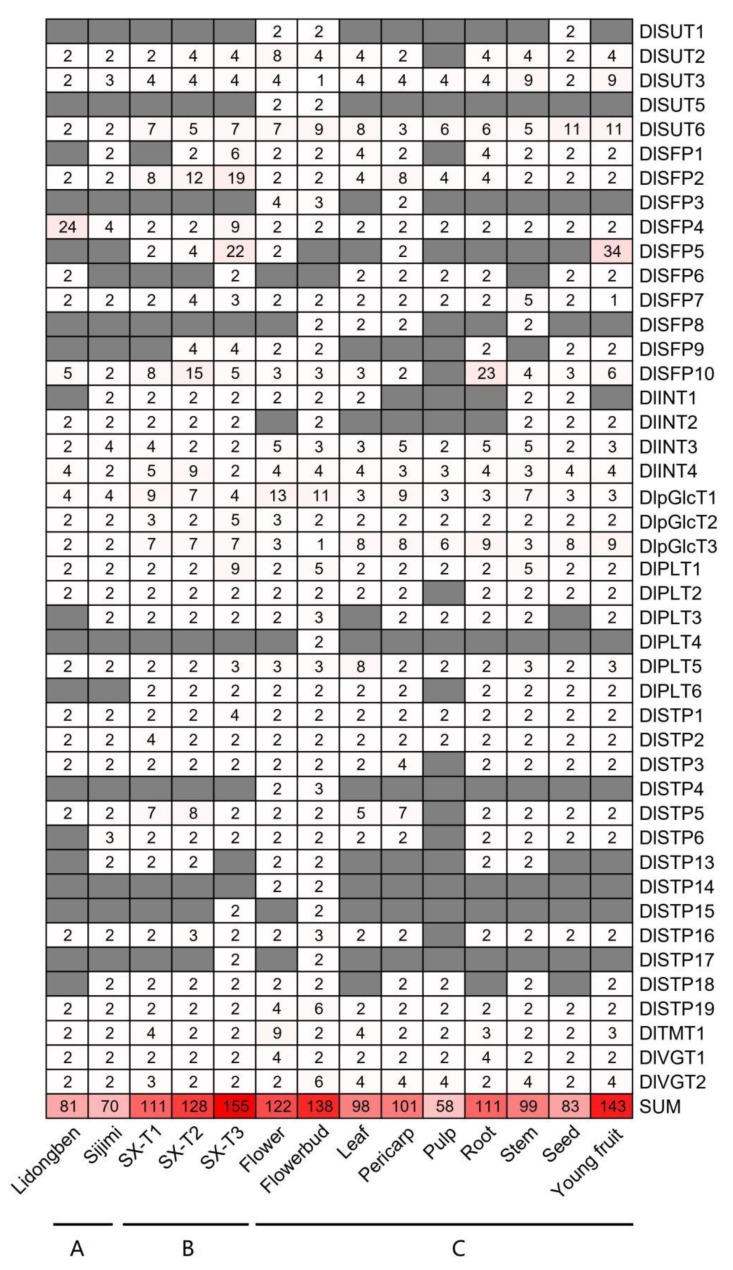
The numbers of AS events of the DlST genes detected in different varieties and tissues. (**A**) The AS events of the DlST genes detected in ‘Lidongben’ and ‘Sijimi’. (**B**) The AS events of the DlST genes detected in different floral bud development stages in ‘Shixia’. SX-T1 represent stage before the emergence of floral primordia, SX-T2 represent stage of the appearance of red dot, and SX-T3 represent stage of the appearance of the first inflorescence apical buds. (**C**) The AS events of the DlST genes detected in nine organs of ‘Sijimi’ clutivar.

Alternative splicing (AS) is commonly found in plant species and have a regulation of gene expression [34]. To study the protein translation of DlST genes, five types of AS events in different longan varieties (‘Lidongben’ and ‘Sijimi’ cultivar), different floral bud development in ‘Shixia’ cultivar, and nine tissues of the ‘Sijimi’ cultivar, including alternative 3′splice site (A3S), alternative 5′splice site (A5S), retained intron (RI), skipped exon (SE), and alternative exon ends (AE) were examined (Figure 6 and Table S2). The numbers of AS events in ‘Lidongben’ is more than that in ‘Sijimi’. The most and least types of AS events among the two cultivars were alternative 5′splice site (60) and skipped exon (4), respectively. Furthermore, AS events existed in 31 DlST genes in ‘Sijimi’, followed by 26 genes in ‘Lidongben’ (Figure 7 and Table S2). Additionally, six genes (*DlSFP1*, *DlINT1*, *DlPLT3*, *DlSTP6*, *DlSTP13* and *DlSTP18*) showed 13 specific AS events in ‘Sijimi’. Interestingly, no skipped exon event exists in ’Sijimi’. Taken together, the specific AS events in ‘Sijimi’ might arrange different proteins to drive various functions.

To investigate the influence of different development stages in AS events of DlST genes, we examined AS events of different floral bud development stage in ‘Shixia’ (Figure 7 and Table S2). A total of 155 AS events was identified in SX-T3 stage, followed by 128 in SX-T2 stage and 111 in SX-T1 stage. In addition, 36 DlST genes displayed AS events in SX-T3 stage, come next 34 in SX-T2 stage and 32 in SX-T1 stage (Figure 7 and Table S2). Furthermore, three genes (*DlSFP6*, *DlSTP15* and *DlSTP17*) underwent specific AS event in SX-T3 stage, and no specific AS event was identified in other developmental stages.

By analyzing the transcriptome of nine tissues in ‘Sijimi’, we found that young fruit (143) and pulp (58) accounted for the largest and smallest number of AS events in DlST genes, respectively. Alternative 5′splice site (143) and skipped exon (58) was the most and least common, respectively. A total of 42 DlST genes underwent AS events in flower buds, with 38 genes in flower and 33 genes in pericarp, stem and young fruit; the AS events in pulp was the lowest (21) (Figure 7 and Table S2). Furthermore, six specific AS events were detected in flower buds (*DlPLT4*, *DlSTP15* and *DlSTP17*). Taken together, AS events may affect flower bud differentiation by forming diverse transcripts.

### 2.6. Transcript Profiles of DlST Genes in Different Plant Tissues

**Figure 8:**
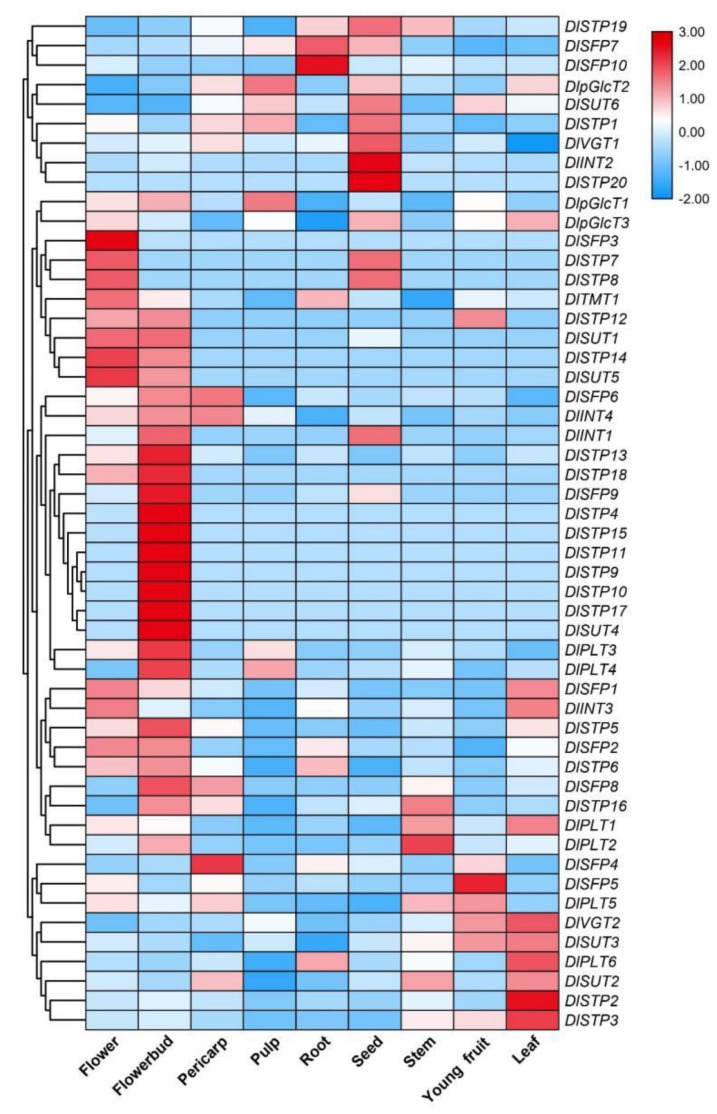
Expression profiles of the DlSTs in different tissues. The color scale represents the log2 (expression values+1); the red and blue colors indicate the higher or lower expression level, respectively.

To elucidate the potential roles of the DlST genes during longan development, we downloaded expression profile data for different tissues from the NCBI database (GSE84467). Nine organs represented in this expression array, including flower bud, flower, leaf, root, stem, seed, pulp, young fruit and pericarp. The results showed that 80.77% (42 of 52) of DlSTs were expressed in flower bud and 73.08% showed expression level in the flower (Figure 8). A total of 65.38%, 63.46%, 61.54% and 59.62% of DlSTs were expressed in the pericarp, stem, seed, root, leaf and young fruit respectively. Approximately 44.23% DlST genes were detected in pulps. Only 42.31% (22 of 52) genes displayed transcript level in all nine tissue in which 23 DlST genes (*DlSFP1*, *DlSFP2*, *DlSFP7*, *DlINT3*, *DlINT4*, *DlpGlcT1*, *DlpGlcT2*, *DlpGlcT3*, *DlPLT1*, *DlPLT2*, *DlPLT5*, *DlPLT6*, *DlSTP1*, *DlSTP2*, *DlSTP3*, *DlSTP16*, *DlSTP19*, *DlTMT1*, *DlVGT1*, *DlVGT2*, *DlSUT2*, *DlSUT3* and *DlSUT6*) exhibited high expression level in at least six longan tissues. On the contrary, *DlSTP17* only showed a significantly low transcript abundence in the flowerbud and nine genes (*DlSFP6*, *DlSTP7*, *DlSTP8*, *DlSTP9*, *DlSTP10*, *DlSTP11*, *DlSTP12*, *DlSTP20* and *DlSUT4*) displayed no expression in all nine tissues.

### 2.7. Transcript Profiles of DlST Genes in Different Longan Varieties and Floralbud Developmental Stages

**Figure 9:**
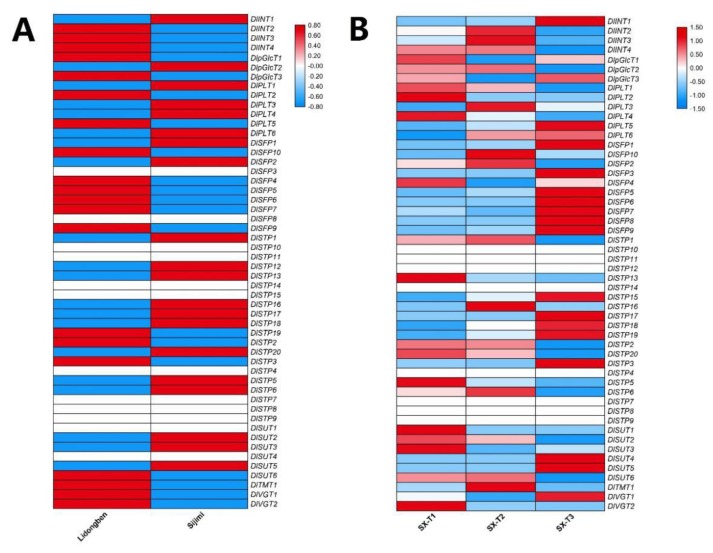
Expression profiles of the DlSTs in different varieties and tissues. (**A**) The expression profile of the DlST genes in ‘Lidongben’ and ‘Sijimi’. (**B**) The expression profile of the DlST genes in different floral bud development stages in ‘Shixia’. SX-T1 represent stage before the emergence of floral primordia, SX-T2 represent stage of the appearance of red dot, and SX-T3 represent stage of the appearance of the first inflorescence apical buds. The color scale represents the log2 (expression values + 1); the red and blue colors indicate the higher or lower expression level, respectively.

‘Lidongben’ and ‘Shixia’ are common longan varieties, while ‘Sijimi’ is a special cultivar exhibiting continuous flowering trait. To study whether the DlST genes affected longan bloom, we compared the transcript levels of DlSTs in two longan varities (Figure 9A). The results showed that 20 DlSTs (*DlINT1*, *DlpGlcT2*, *DlPLT1*, *DlPLT3*, *DlPLT4*, *DlPLT6*, *DlSFP1*, *DlSFP2*, *DlSTP1*, *DlSTP12*, *DlSTP13*, *DlSTP16*, *DlSTP17*, *DlSTP18*, *DlSTP20*, *DlSTP5*, *DlSTP6*, *DlSUT2*, *DlSUT3* and *DlSUT5*) showed higher transcript abundence in ‘Sijimi’ than that in ‘Lidongben’. Furthermore, a total of six genes (*DlSTP1*, *DlINT1*, *DlSUT2*, *DlSFP1*, *DlSTP5* and *DlSTP6*) belong to the different expression gene (DEG).

To analyze whether the DlST plays a role in flower bud differentiation, we compared the transcript levels of DlSTs during floral bud development in ‘Shixia’ (Figure 9B). The results showed that there were 18 DlSTs (*DlINT2*, *DlINT3*, *DlINT4*, *DlpGlcT2*, *DlPLT1*, *DlPLT2*, *DlPLT4*, *DlSFP2*, *DlSTP1*, *DlSTP13*, *DlSTP2*, *DlSTP20*, *DlSTP5*, *DlSTP6*, *DlSUT2*, *DlSUT6*, *DlTMT1* and *DlVGT2*) showed higher transcript levels in T1 and T2 stage. Taken together, ten genes (*DlpGlcT2*, *DlPLT1*, *DlPLT4*, *DlSFP2*, *DlSTP1*, *DlSTP13*, *DlSTP20*, *DlSTP5*, *DlSTP6* and *DlSUT2*) showed higher expression in both analyses, suggesting that these genes may function in flower bud differentiation.

### 2.8. Expression Profiles of DlST Genes at Fruit Development Stages of Longan

**Figure 10:**
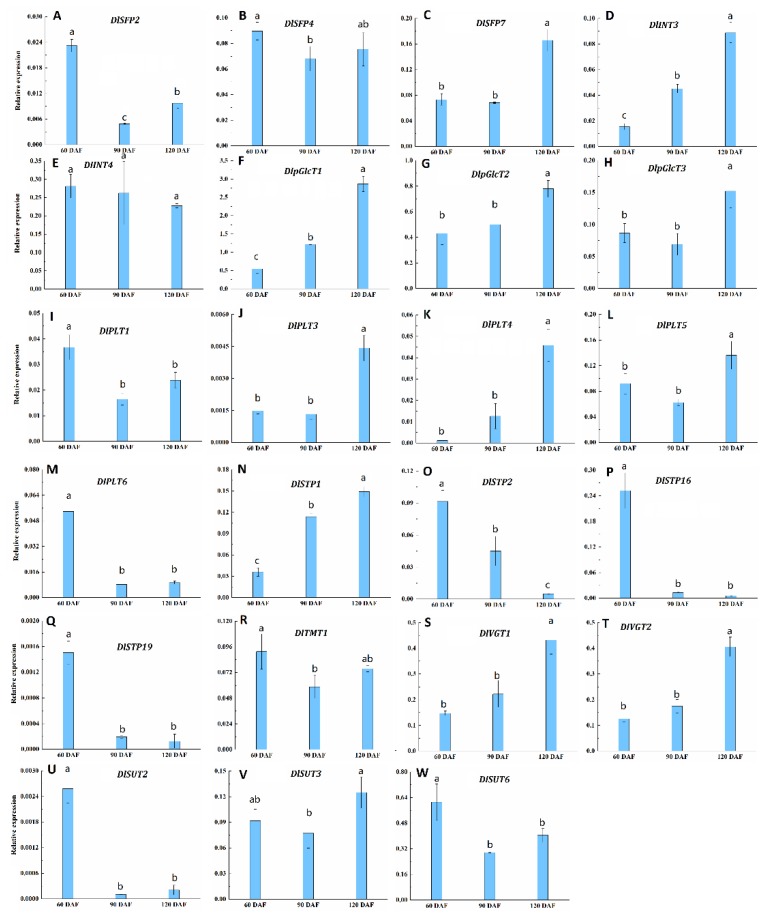
Expression profiles of 23 DlST genes during longan fruit development. Values were mean ± standard error (SE) (*n* = 3). Lowercase letter(s) above the bars indicate significant differences (*P*-value = 0.05, LSD) among different developmental stages.

Sweetness, determined by sugar composition and content, is a key factor to determine the quality of longan fruits. The main sugar components in longan fruit are sucrose, glucose, and fructose. To further study the potential roles of DlSTs in longan fruit sugar accumulation, we selected 23 genes that might be related to sugar accumulation for qRT-PCR analysis during longan fruit development (Figure 10). The results showed that the transcript abundance of 11 genes (*DlSFP2*, *DlSFP4*, *DlINT4*, *DlPLT1*, *DlPLT6*, *DlSTP2*, *DlSTP16*, *DlSTP19*, *DlTMT1*, *DlSUT2* and *DlSUT6*) was higher at 60 DAF than other developmental stages; besides, other 12 genes (*DlSFP7*, *DlINT3*, *DlpGlcT1*, *DlpGlcT2*, *DlpGlcT3*, *DlPLT3*, *DlPLT4*, *DlPLT5*, *DlSTP1*, *DlVGT1*, *DlVGT2* and *DlSUT3*) showed higher expressional values at 120 DAF than other stages.

For the *DlSFP* subfamily, the expression of *DlSFP2* declined slightly from 60 to 90 DAF and then showed a slight increasing from 90 to 120 DAF. The *DlSFP7* transcript showed a stable value from 60 to 90 DAF and then increase toward fruit maturity. The *DlSFP4* presented high expression level at 60 DAF, then slightly declined from 60 to 90 DAF and increased slightly from 90 to 120 DAF. For the *DlINT* clade, the relative expression trend of the two *DlINT* genes (*DlIN3* and *DlINT4*) are opposite. The expression of *DlIN3* and *DlIN4* showed up regulation and down regulation during fruit development, respectively. For the *DlpGlcT* subfamily, *DlpGlcT1* and *DlpGlcT*2 transcript showed persistent up regulation from 60 DAF to 120 DAF, the transcript level of *DlpGlcT*3 was declined from 60 to 90 DAF, but increased afterward as fruit matured. In *DlPLT* subfamily, the transcript abundance of *DlPLT1* and *DlPLT5* showed the highest level at 60 DAF, then declined slightly from 60 to 90 DAF and increased slightly from 90 to 120 DAF. *DlPLT4* showed up regulation through fruit development. *DlPLT3* remained a stable expression level from 60 to 90 DAF and showed up regulation from 90 to 120 DAF. *DlPLT6* showed down regulation from 60 to 90 DAF and remained unchanged from 90 to 120 DAF. For the *DlSTP* subfamily, The *DlSTP2*, *DlSTP16* and *DlSTP19* showed a continuous decline expression toward fruit maturity. However, the expression level of *DlSTP1* revealed constant increment toward fruit ripening. For the *DlTMT* subfamily, *DlTMT1* down regulated from 60 to 90 DAF then increased. For the *DlVGT* subfamily, *DlVGT1* and *DlVGT2* were both up regulated from 60 to 120 DAF. For the *DlSUT* subfamily, *DlSUT2* and *DlSUT6* markedly decreased from 60 to 90 DAF then slightly increased. *DlSUT3* showed a slight decrease from 60 to 90 DAF but further increased markedly at 120 DAF.

## 3. Discussion

### 3.1. Identification, Phylogenetic and Structural Analysis of the DlSTs

The search against *Dimocarpus longan* genome has identified 52 sugar transporters, including six SUTs and 46 MSTs, suggesting that SUT is a very small gene family among sugar transporter families. The similar results were observed in other plants, for instance, six SUTs were identified in pear [25], five in rice [21], four in grapevine [23], and three in tomato [20] and Chinese jujube [27]. Furthermore, the number of MSTs in longan (46) is smaller than that in *Arabidopsis* (53) [19], grapevine (61) [23], rice (65) [22], strawberry (58) [24], pear (69) [25], apple (64) [26], Chinese jujube (53) [27] and tomato (49) [20]. Phylogenetic analysis indicated that DlSTs could group into two distinct clades (sucrose and monosaccharide transporters), furthermore, MST proteins divided into seven different groups (Figure 2). The result in consistent with previous reported in other plants, such as *Arabidopsis*, tomato, grape, and rice [20,22,23], indicating a reliable and reasonable result of classification of longan MST families. As displayed in Figure 3, different subfamilies have similar conserved domains, indicating the same function among the same subfamily members. We also compared the different subfamily members in nine plants (Table 2). Interestingly, as in rice, tomato, strawberry, apple and Chinese jujube, STPs form the largest subfamilies in longan. In addition, exon–intron organization analysis indicated that DlST genes have different exon numbers, arranging from one to 23 (Figure 2), a similar result was obtained from tomato [20].

### 3.2. Cis-Elements in the DlST Promoters

*Cis*-acting elements are essential in many biological processes and stress responses [35]. In this study, several common motifs were identified in DlST promoter regions, for instance, *cis*- elements involved in light responsiveness. In addition, DlST genes may play a role in circadian regulation because of the existence of the circadian element.

However, there are no common cis-elements detected in the DlSTs promoter, which is different from previous findings in grape and strawberry, indicating a different transcriptional regulation mechanism of ST genes in different species. A total of seven unique cis-elements were identified, which is smaller than that in pear and strawberry. Intriguingly, two of the seven cis-elements were only discovered in the *DlpGlcT3* promoter, and only one in *DlSUC2*, *DlPLT3*, *DlSTP19*, *DlPLT5* and *DlSTP12*, respectively. DlST promoters carry different cis-elements that may explain differential expression. Additional analysis should be carried on to test this hypothesis.

### 3.3. AS events Exist in the Longan ST Gene Family

RNA-Seq show more benefits than gene expression microarrays, such as broad dynamic range, high sensitivity and accuracy, ability to discover novel genes and alternative splicing (AS), which involves the conversion of precursor mRNA into mature mRNA. AS event exists in the great mass of eukaryotic protein-coding genes and considered to be a key regulatory mechanism, which increase the diversity of transcriptome and proteome and adaptation during plant evolution and stress [36,37]. AS is commonly found in varies plants, such as *Arabidopsis* [38], rice [39] and soybean [40]. To date, there are few reports related to the changing of AS events for ST genes. Although the numbers of AS events in ‘Lidongben’ is more than that in ‘Sijimi’, however, more ST genes in ‘Sijimi’ had AS events. Among the nine organs, the numbers of genes, which underwent AS events, is more in flower bud than that in other tissues. Additionally, six specific AS events were identified in flower bud. Taken together, DlSTs may contribute to longan bloom by changing gene expression levels via variety of AS events, which. All of these results require further validation to confirm the effect of alternative splicing in ST gene function.

### 3.4. Expression of Sugar Transporters during Longan Fruit Development

Sucrose markedly increased during longan fruit development, and that its concentration was significantly higher than glucose and fructose when fruits getting maturity (Figure 1). Furthermore, sucrose is a universal form of long-distance carbon transport in most plants [41,42]. However, although SUT may not be essential for phloem loading, but serve as proton co-transporters for phloem unloading [26]. A total of six SUTs were identified in longan and only three genes (*DlSUT2*, *DlSUT3* and *DlSUT6*) expressed in pulp. Although the expression level of *DlSUT2* was lower than *DlSUT3* and *DlSUT6* (Figure 10), *DlSUT2* showed high transcriptional level in leaves, young fruit and stems (Figure 8), suggesting that *DlSUT2* may function in sucrose transport from source to sink. Many polyol transporters have been found in plants, where they are responsible for the polyols loading [43,44,45,46,47]. Six polyol transporters were identified in *Arabidopsis thaliana*, which belong to non-polyol-translocating species but their physiological role is still poorly understand [48,49,50]. Our results indicate six polyol transporter genes exsit in longan genome and five DlPLTs were expressed in pulp but showed relative lower expression levels. However, polyols are not the transported sugar in the phloem of longan, the role of these transporters is far from being clear.

For fruits, most of the soluble sugars are stored in the central vacuoles. As a result, vacuoles play a critical role in plant growth and development [51,52]. This process requires sugars which transported from the cytosol by carrier proteins localized on the tonoplast membrane, such as vGT [53], TMT [54], SFP [55]. We found two DlvGTs, one TMT and ten SFPs in longan genome, respectively. However, only six genes (*DlSFP2*, *DlSFP4*, *DlSFP7*, *DlTMT1*, *DlVGT1* and *DlVGT2*) were expressed in pulp. Furthermore, the expression of *DlVGT1* and *DlVGT2* was high in fruit and in accordance with the sucrose increase, suggesting their important roles in sugar accumulation. Monosaccharides, which were transported into fruits, not only play important roles in the increasing of the monosaccharide content, but also in providing substrates for sucrose synthesize [56]. The monosaccharide transporters *DlpGlcT1*, *DlpGlcT2* and *DlSTP1*, which showed up-regulated trends during the fruit development stages (Figure 10), should be considered as targets for fruit sugar accumulation.

## 4. Materials and Methods

### 4.1. Plant Materials

*Dimocarpus longan* cv ‘Songfengben’ plants were used in present study and grown under the standard cultivation conditions at Fuzhou Longan and Loquat Resource Nurseries of National Fruit Gene-Pool, Fuzhou, China. The fruit samples were collected at 60, 90, and 120 days after flowering (DAF), respectively. A total of 60 fruits were picked from one tree at each developmental stage. In addition, 60 fruits divided into three biological replicates in which containing 20 fruits. After the peel and seed coat removed, the pulp was immediately frozen in liquid nitrogen and stored at −80 °C until used.

### 4.2. Extraction and High Performance Liquid Chromatography (HPLC) Analysis of Soluble Sugar

The extraction and HPLC analysis of sucrose, fructose and glucose in pulp was performed as described by according to the method by Fang et al. [57]. The average content was expressed in grams per kilogram of fresh weight (g kg^−1^ FW).

### 4.3. RNA Isolation and Quantitative Real-Time Reverse Transcription PCR (qRT-PCR) Analysis

Total RNA was isolated using a standard RNAprep pure Plant kit (Tiangen, Beijing, China) according to the recommended protocol. The amount and quality of the total RNA was confirmed using a NanoDrop 2000 spectrophotometer (Thermo Scientific, Waltham, MA USA). One microgram of purified RNA was reverse transcribed into cDNA using TransScript One-Step gDNA Removal and cDNA Synthesis SuperMix (TRANS, Beijing, China) following the manufacturer’s protocol. qRT-PCR was performed using SYBR Green I Master Mix (Takara, Dalian, China) and the expression levels of ST genes were normalized to the longan *Actin1* gene (Dlo_028674) [58]. qRT-PCR was performed on a LightCycler 96 Real-Time PCR Systems (Roche, NC, USA). Primer sequences for qRT-PCR analysis are listed in Table S3.

### 4.4. Identification of ST Family in Longan

Longan ST gene family were identified by performing a BLASTP analysis of the 62 *Arabidopsis* ST genes against the longan genome [59] with E-value 1 × 10^−5^. The 62 *Arabidopsis* ST proteins were gained from the previous study and the *Arabidopsis* database (http://www.arabi-dopsis.org/) [20,60]. Finally, a total of 52 longan STs (DlSTs) were identified for further analysis.

### 4.5. Phylogenetic Analysis of DlSTs

ClustalX program was used to make multiple sequence alignments. A phylogenetic analysis was performed using MEGA6 program (https://www.megasoftware.net/) employing the neighbor-joining (NJ) method [61] with a bootstrap value 1000.

### 4.6. Conserved Motifs, Gene Structure and Promoter Motifs of DlST Genes

Motifs of DlST proteins was analyzed using Multiple EM for Motif Elicitation (MEME) (http://meme.nbcr.net/meme/cgi-bin/meme.cgi) to confirm the conserved motifs [62]. The relative parameters were set as: maximum number of motifs, 600; number of repetitions, any; optimum width, 15–60; and maximum number of motifs, 15. The gene structure display server 2.0 (GSDS, http://gsds.cbi.pku.edu.cn) was used to analyze the constituents of the exons/introns of the DlST genes [63]. The cis-motifs of DlST promoters were identified in a 2-kb upstream coding sequence of DlST genes. Discovered motifs were analyzed using PLACE (https://www.dna.affrc.go.jp/PLACE/?action=newplace).

### 4.7. Identification of Alternative Splicing (AS) Events

The discovery the AS events of DlSTs, we use three transcriptome databases, including one for different longan varieties (‘Sijimi’ and ‘Lidongben’) [64], one for different floral bud development stages in ‘Shixia’ cultivar [65], and one ‘Sijimi’ transcriptome database containing nine longan tissues [59]. The quality of RNA-seq data are listed in Table S4. The AS events were identified by the software Asprofile [66].

### 4.8. Expression Analysis of DlSTs in Different Longan Varieties and Tissues

To study the transcriptional accumulation of *DlST* genes in different longan varieties and tissues, the three transcriptome datasets mentioned above were used. The fragments per kilo base of the exon model per million mapped values (FPKM) were log2-transformed and normal standardization according to row scale, and heat maps were exhibited using the software TBtools [67].

### 4.9. Statistical Analyses

The data analyses were conducted by SPSS statistics 21.0 (IBM Inc., NY, USA) and graphed with Origin 9.1 software. ANOVA was chosen to test the difference between the means of different stages (*P*-value = 0.05).

## 5. Conclusions

In summary, 52 *DlSTs* were discovered in *Dimocarpus longan* genome, and they were divided into eight clades. Different species have different transcriptional regulation in the *DlST* gene family. AS events analyses indicated that several *DlST* genes may play a role in longan flower bud differentiation. Expression profile analyses suggested ten and seven *DlSTs* may play key role in longan bloom and sugar accumulation. Our results help to further understanding the complicated functions of sugar transporter genes in longan and other woody plants.

## Figures and Tables

**Table 1:** Characteristics of 52 DlST genes in longan.

Gene ID	Locus	Location	Strand	Genomic (bp)	cDNA (bp)	Protein (aa)	TMD ^a^	Subcelular Localizations ^b^
*DlSUT1*	Dlo_026383.1	scaffold60:1076732..1078932	-	2201	1521	506	12	plas: 6, vacu: 4, golg: 2, cyto: 1, mito: 1
*DlSUT2*	Dlo_026385.1	scaffold60:1111276..1114774	-	3499	1539	512	12	plas: 7, vacu: 4, cyto: 1, mito: 1, golg: 1
*DlSUT3*	Dlo_026249.1	scaffold6:2022196..2030269	+	8074	1818	605	11	plas: 9, E.R.: 5
*DlSUT4*	Dlo_026381.1	scaffold60:1059167..1063033	-	3867	1446	481	11	vacu: 9, plas: 4, cyto: 1
*DlSUT5*	Dlo_000769.1	scaffold1001:62604..64968	+	2365	1341	446	10	plas: 9, chlo: 2, vacu: 2, E.R.: 1
*DlSUT6*	Dlo_027597.1	scaffold656:41881..49342	-	7462	1746	581	14	plas: 12, golg: 2
*DlSFP1*	Dlo_032238.1	scaffold875:109482..113458	-	3977	1461	486	11	plas: 12, vacu: 1, E.R.: 1
*DlSFP2*	Dlo_022839.1	scaffold498:125274..129519	-	4246	1332	443	11	plas: 13, vacu: 1
*DlSFP3*	Dlo_020112.1	scaffold407:102424..107968	+	5545	1419	472	12	plas: 6, vacu: 4, golg: 3, E.R.: 1
*DlSFP4*	Dlo_020136.1	scaffold407:307933..318617	-	10,685	1962	653	14	plas: 10, E.R.: 2, nucl: 1, vacu: 1
*DlSFP5*	Dlo_020110.1	scaffold407:79196..84320	+	5125	1479	492	12	plas: 7, vacu: 5, E.R.: 1, golg: 1
*DlSFP6*	Dlo_020111.1	scaffold407:94672..100310	+	5639	1365	454	11	plas: 10, E.R.: 2, cyto: 1, vacu: 1
*DlSFP7*	Dlo_025827.1	scaffold59:1357215..1361965	-	4751	1461	486	12	plas: 8, golg: 3, vacu: 2, E.R.: 1
*DlSFP8*	Dlo_032237.1	scaffold875:100632..103833	-	3202	1137	378	8	plas: 11, nucl: 1, vacu: 1, E.R.: 1
*DlSFP9*	Dlo_000038.1	scaffold1:326041..331429	+	5389	1407	468	10	plas: 7, vacu: 3, golg: 3, E.R.: 1
*DlSFP10*	Dlo_020109.3	scaffold407:56975..68551	+	11,577	1602	533	12	plas: 8, golg: 4, vacu: 2
*DlINT1*	Dlo_020864.1	scaffold43:1222304..1226297	+	3994	1737	578	10	plas: 10, E.R.: 2, nucl: 1, vacu: 1
*DlINT2*	Dlo_020865.1	scaffold43:1231067..1233489	+	2423	1731	576	12	plas: 11, E.R.: 2, nucl: 1
*DlINT3*	Dlo_020216.1	scaffold41:171298..173058	-	1761	1509	502	10	vacu: 7, plas: 3, cyto: 1, mito: 1, extr: 1, E.R.: 1
*DlINT4*	Dlo_027030.1	scaffold633:93770..101553	-	7784	1527	508	12	vacu: 8, golg: 3, plas: 2, E.R.: 1
*DlpGlcT1*	Dlo_016844.1	scaffold322:288796..294168	-	5373	1584	527	10	plas: 6, vacu: 6, E.R.: 1, golg: 1
*DlpGlcT2*	Dlo_001687.1	scaffold1079:68275..75345	+	7071	1650	549	11	plas: 8, E.R.: 4, chlo: 1, vacu: 1
*DlpGlcT3*	Dlo_011515.2	scaffold2146:12441..18212	+	5772	1728	575	11	plas: 12, chlo: 1, E.R.: 1
*DlPLT1*	Dlo_023817.1	scaffold529:18313..22834	+	4522	1620	539	10	plas: 10, E.R.: 2, cyto: 1, vacu: 1
*DlPLT2*	Dlo_000074.1	scaffold1:627464..630485	-	3022	1521	506	12	vacu: 8, plas: 3, golg: 2, cyto: 1
*DlPLT3*	Dlo_002670.1	scaffold1137:86368..88138	-	1771	1626	541	12	plas: 6, vacu: 4, golg: 3, E.R.: 1
*DlPLT4*	Dlo_002669.1	scaffold1137:83579..84955	-	1377	1377	458	11	vacu: 8, plas: 4, golg: 2
*DlPLT5*	Dlo_011139.1	scaffold21:402299..405393	-	3095	1536	511	11	plas: 7, vacu: 3, golg: 3, E.R.: 1
*DlPLT6*	Dlo_004484.1	scaffold1293:64280..65671	-	1392	1392	463	10	details plas: 7, vacu: 7
*DlSTP1*	Dlo_003645.1	scaffold122:80918..82578	+	1661	1413	470	11	plas: 9, vacu: 2, golg: 2, E.R.: 1
*DlSTP2*	dlo_035245.1	scaffold796:47859..50056	+	2198	1503	501	11	plas: 11, vacu: 2, cyto: 1
*DlSTP3*	Dlo_027739.1	scaffold66:580548..585736	-	5189	1578	525	12	vacu: 8, plas: 2, E.R.: 2, cyto: 1, golg: 1
*DlSTP4*	Dlo_002303.1	scaffold110:684604..686707	+	2104	1560	519	11	vacu: 6, plas: 4, cyto: 1, mito: 1, extr: 1, E.R.: 1
*DlSTP5*	Dlo_033294.1	scaffold93:799728..804065	-	4338	1539	512	10	vacu: 12, cyto: 1, plas: 1
*DlSTP6*	Dlo_033296.1	scaffold93:820613..826961	-	6349	1767	588	10	plas: 9, vacu: 2, cyto: 1, mito: 1, E.R.: 1
*DlSTP7*	dlo_036328.1	scaffold115:387758..389514	-	1757	1494	498	12	plas: 8, vacu: 5, cyto: 1
*DlSTP8*	dlo_035834.1	scaffold115:438581..440337	-	1757	1494	498	12	plas: 8, vacu: 5, cyto: 1
*DlSTP9*	dlo_038438.1	scaffold115:350084..351885	-	1802	1539	513	12	plas: 9, vacu: 3, cyto: 1, golg: 1
*DlSTP10*	dlo_037985.1	scaffold115:387728..389529	-	1802	1539	513	12	plas: 9, vacu: 3, cyto: 1, golg: 1
*DlSTP11*	dlo_036073.1	scaffold115:438551..440352	-	1802	1539	513	12	plas: 9, vacu: 3, cyto: 1, golg: 1
*DlSTP12*	dlo_035972.1	scaffold115:327583..329355	-	1773	1509	503	12	plas: 10, E.R.: 2, cyto: 1, golg: 1
*DlSTP13*	Dlo_033293.1	scaffold93:773962..780790	-	6829	1689	562	10	plas: 9, golg: 3, vacu: 1, E.R.: 1
*DlSTP14*	Dlo_009764.1	scaffold1902:44598..46993	+	2396	1494	497	12	vacu: 9, plas: 4, cyto: 1
*DlSTP15*	Dlo_029426.1	scaffold73:1401902..1404530	+	2629	1551	516	12	plas: 5, vacu: 5, golg: 2, cyto: 1, mito: 1
*DlSTP16*	Dlo_011195.1	scaffold21:967674..970706	+	3033	1560	519	12	plas: 4, vacu: 3, E.R.: 3, chlo: 1, cyto: 1, pero: 1, golg: 11
*DlSTP17*	Dlo_032047.1	scaffold860:122653..124652	-	2000	1542	513	12	plas: 10, golg: 2, cyto: 1, vacu: 1
*DlSTP18*	Dlo_027736.1	scaffold66:557824..560794	+	2971	1395	464	11	plas: 12, vacu: 1, E.R.: 1
*DlSTP19*	Dlo_007964.1	scaffold1654:30425..34897	-	4473	1500	499	12	vacu: 13, cyto: 1
*DlSTP20*	Dlo_027737.1	scaffold66:563003..574149	+	11,147	1620	539	10	plas: 7, vacu: 5, cyto: 1, golg: 1
*DlTMT1*	Dlo_028299.1	scaffold69:577583..582174	+	4592	2226	741	10	plas: 12, vacu: 1, golg: 1
*DlVGT1*	Dlo_017615.1	scaffold34:1130206..1139211	-	9006	1476	491	12	vacu: 9, plas: 3, golg: 2
*DlVGT2*	Dlo_029523.2	scaffold73:2182255..2185057	+	2803	1437	478	11	plas: 11, vacu: 3

^a^ The number of transmembrane domains (TMDs) was predicted by TMHMM Server v2.0 (http://www.cbs.dtu.dk/services/TMHMM/) [32]. ^b^ The subcellular localizations were predicted by WoLFPSORT (http://wolfpsort.org/) [33]. plas, plasma membrane; vacu, vacuolar membrane; chlo, chloroplast; mito, mitochondrion; nucl, nucleus; ER, endoplasmatic reticulum; cyto, cytosol; golg, golgi.

**Table 2:** Comparative analysis of sugar transporter (ST) gene families in *Arabidopsis*, rice, strawberry, pear, tomato, grapevine, Chinese jujube and apple.

	No. of Genes								
Subfamily	Longan	*Arabidopsis*	Rice	Strawberry	Pear	Tomato	Grapevine	Chinese Jujube	Apple
SUC	6	9	5	8	6	3	4	3	9
STP	20	14	29	24	20	18	22	16	30
VGT	2	3	2	2	3	2	2	2	3
PLT	6	6	15	7	23	8	5	10	10
INT	4	4	3	3	6	4	3	5	4
TMT	1	3	6	3	6	3	3	3	5
SFP	10	19	6	16	5	10	22	14	8
pGlcT	3	4	4	3	6	4	4	3	4
Toatl	52	62	70	66	75	52	65	56	73

## Supplementary Materials

The following are available online at https://www.mdpi.com/2223-7747/9/3/342/s1, Table S1: Analysis and distribution of conserved motifs in Dimocarpus longan DlST proteins. Table S2: Total number of AS events detected in differenft longan varieties and tissues. Table S3: The primer sequences of 23 DlSTs and reference gene in longan. Table S4:The summary of RNA-seq data.
